# The Skeletal Organic Matrix from Mediterranean Coral *Balanophyllia europaea* Influences Calcium Carbonate Precipitation

**DOI:** 10.1371/journal.pone.0022338

**Published:** 2011-07-22

**Authors:** Stefano Goffredo, Patrizia Vergni, Michela Reggi, Erik Caroselli, Francesca Sparla, Oren Levy, Zvy Dubinsky, Giuseppe Falini

**Affiliations:** 1 Dipartimento di Chimica ‘G. Ciamician’, Alma Mater Studiorum Università di Bologna, Bologna, Italy; 2 Marine Science Group, Alma Mater Studiorum Università di Bologna, Bologna, Italy; 3 Dipartimento di Biologia Evoluzionistica Sperimentale, Alma Mater Studiorum Università di Bologna, Bologna, Italy; 4 The Mina and Everard Goodman Faculty of Life Sciences, Bar Ilan University, Ramat Gan, Israel; Consejo Superior de Investigaciones Cientificas, Spain

## Abstract

Scleractinian coral skeletons are made mainly of calcium carbonate in the form of aragonite. The mineral deposition occurs in a biological confined environment, but it is still a theme of discussion to what extent the calcification occurs under biological or environmental control. Hence, the shape, size and organization of skeletal crystals from the cellular level through the colony architecture, were attributed to factors as diverse as mineral supersaturation levels and organic mediation of crystal growth. The skeleton contains an intra-skeletal organic matrix (OM) of which only the water soluble component was chemically and physically characterized. In this work that OM from the skeleton of the *Balanophyllia europaea*, a solitary scleractinian coral endemic to the Mediterranean Sea, is studied *in vitro* with the aim of understanding its role in the mineralization of calcium carbonate. Mineralization of calcium carbonate was conducted by overgrowth experiments on coral skeleton and in calcium chloride solutions containing different ratios of water soluble and/or insoluble OM and of magnesium ions. The precipitates were characterized by diffractometric, spectroscopic and microscopic techniques. The results showed that both soluble and insoluble OM components influence calcium carbonate precipitation and that the effect is enhanced by their co-presence. The role of magnesium ions is also affected by the presence of the OM components. Thus, *in vitro*, OM influences calcium carbonate crystal morphology, aggregation and polymorphism as a function of its composition and of the content of magnesium ions in the precipitation media. This research, although does not resolve the controversy between environmental or biological control on the deposition of calcium carbonate in corals, sheds a light on the role of OM, which appears mediated by the presence of magnesium ions.

## Introduction

Organisms exert an exceptional control over the polymorphism, orientation and morphology of their mineral components through a series of biochemical processes generally included under the term biomineralization [1]–[4]. It is generally recognised that the biomineralization process involves several steps: the fabrication of a hydrophobic solid organic substrate; the nucleation of crystalline materials associated with specific polyanionic macromolecules that cover the internal wall of the organic scaffold; the crystal growth, controlled by new secretions of polyanionic macromolecules; the termination of the process by secretion of inhibitory macromolecules [1], [5].

Specific functions related to different types of molecules were shown for the organic matrix (OM) associated to the calcium carbonate polymorphs found in animal skeletal and shell elements: calcite, aragonite, vaterite or amorphous calcium carbonate (ACC). Pioneering *in vitro* experiments have shown the influence of acidic glycol-proteins on the morphological control of calcite deposition, highlighting an important role of the glycosidic regions in morphology modulation [6]. The capability of OM to determine the aragonite versus calcite polymorphic selection was determined in mollusc shells [7]. This organic control on the calcium carbonate polymorphism was also verified by the abrupt transition calcite-aragonite in abalone shells, accompanied by the synthesis of specific polyanionic proteins [8]. Acidic macromolecules associated with aragonite or vaterite from fish otoliths influence this polymorphic selection [9]. Families of mixed acidic macromolecules have always been used in all these studies. Only recently a specific protein, Pif1, able to selectively deposit aragonite on a chitin substrate was completely characterized [10]. The function of acid macromolecules can be triggered by the presence of magnesium ions. In a recent research was shown that ACC stabilized by magnesium ions can be converted into calcite by the addition of aspartic acid. The aspartic acid destabilizes the hydration of magnesium ions, thus favouring the precipitation of calcite. This important observation was used to explain how organisms can control the phase transformation from ACC into calcite [11].

Among calcium carbonate depositing organisms, corals are of primary importance due to their dominance in shallow shores in the tropics. Corals give the opportunity of investigating many phenomena of geochemical, biochemical, mineralogical, ecological and paleontological interest [12], [13]. Nowadays, corals relevance as calcium carbonate crystallizers is of extreme importance in view of the effects of global warming and ocean acidification [14], [15].

The mineralogy of aragonitic skeleton of scleractinian corals was investigated in great detail [16], [17]. The building blocks of the skeleton are formed of thin aragonite crystals or fibres (0.04–0.05 µm in diameter), which set up in a tri-dimensional structure. The fibres are preferentially orientated along the crystallographic *c*-axis of aragonite and are assembled as *spherulites*, grouped into fishead-shaped bundles. A building block grow into a vertical spine called *trabecula*; groups of trabeculae form the *septa*, the primary macroscopic structure of the coral skeleton, arranged inside the skeleton in a radial way, which is species specific [18]. At the centre of each spine there is the *centre* of *calcification* (COC, nucleation centre), from which the aragonite fibres growth. Inside the COCs granular sub-micronic crystals grouped in 2–4 µm nuclear packets are located [19], [20].

The morphological relationship between OM and mineral phases in coral unit crystals is poorly understood. OM is synthesized by cells of the calicoblastic epithelium and then secreted into the adjacent subepithelial space in which calcification takes place [21]. The OM of scleractinian corals was investigated in detail in its structure and relation with the mineral phase [20], [22]–[26]. The whole OM is made of glycoproteins in which the proteic regions assume preferentially a α-helical conformation in all the coral species studied, except in one case [26], where a β-sheet conformation was observed. The amino-acid analysis of OM from representative coral skeletons showed a similar composition characterized by a high content of Glu, Asp and Gly. Moreover, Gln, Ser, and Thr were demonstrated to act respectively as N- and O- linking sites for glucidic regions, which were found to be sulphated [24], [27]. X-ray Absorption Near Edge Structure (XANES) mapping of the earliest COC and the fibrous zone, the two main structural entities of the coral skeleton, has shown the correspondence between S-polysaccharides and the spatial arrangement of mineral growth and have evidenced 1) a biochemical zonation that corresponds to the step by step growth, and 2) the general coordination by polyp physiology [20], [22], [24], [26].

The calcium carbonate precipitation is influenced by environmental conditions [28], [29]. It was suggested that seawater Mg/Ca molar ratio influences the polymorphism of calcium carbonate in coral skeletons. Corals should calcify calcite when the Mg/Ca molar ratio is below 2, and aragonite when the ratio is above 2. A cretaceous scleractinians had a calcitic skeleton, when the inferred Mg/Ca ratio of seawater was below 2 [30]. Current Mg/Ca molar ratio of seawater is around 5, then several studies claim that living scleractinians have aragonitic skeletons, due to a strong if not an exclusively environmental control on calcification [31]–[33]. Other researchers have suggested a minor, or indirect, role of the organism in the precipitation processes. In this case the biological control is directed towards the calcium carbonate supersaturation level [34], [35]. However, this is in contrast with the observation of a high biological control on the kind of skeletal material produced in corals [20], [22]–[26], [36], gorgonians [37], molluscs [7], [8], seastars [38] and fish otoliths [9].

Several clues support the existence of a biological influence over the mineral deposition. First, the ultra-structural organization of the aragonite crystals in coral skeleton shows differences from the one of aragonite precipitated abiologically and is species specific. Second, in the coral skeleton, COCs rich of biological macromolecules were identified, suggesting a controlled release of macromolecules in space and time by the organism [20], [22]–[26], [36]. Third, many organisms exert a tremendous biological control over the calcium carbonate polymorphic selection. Indeed, in several mineralized tissues aragonite, calcite and vaterite, the anhydrous calcium carbonate polymorphs, are present and localized in different regions and are never mixed together [1]. This high level of control is mainly due to specific families of acidic macromolecules [1]–[4], which are intimately associated with the mineral phases. Over the years this was demonstrated, mainly by *in vitro* experiments, for mollusc shells and fish otoliths among many (e.g. [7]–[9]).

In the present work, *in vitro* mineralization of calcium carbonate was conducted in presence of both water soluble and insoluble fractions of the intra-skeletal OM from the aragonitic skeleton of *Balanophyllia europaea* (Scleractinia) and magnesium ions at different concentrations. This research was done with the aim to explore the relative biological (OM) and environmental (Mg ions) influence on polymorphism and morphology of CaCO_3_ in the biomineralization of *B. europaea*, an endemic Mediterranean coral living in shallow water (maximum population density at depth <10 m) [39], which has served as model organism and extensively studied in its main aspects of growth, population structure and dynamics, and reproductive biology [40].

## Materials and Methods

### Coral skeletons

Samples of *Balanophyllia europaea* were randomly collected during scuba diving at 5–7 m depth from two sites in the North-Western Mediterranean Sea: Calafuria 43°27′N, 10°21′E (CL) and Elba Island, 42°45′N, 10°24′E (LB). After collection the corals were dipped in a sodium hypochlorite solution (commercial) for 4 days until the polyp tissue was completely dissolved, then the remaining skeletons were washed with double distilled water and dried in oven at 37°C for 24 hr and stored. Each skeleton was analyzed under a binocular microscope to remove fragment of substratum and calcareous deposit produced by other organisms. Successively, the skeletons were ground in a mortar to obtain a fine and homogeneous powder. The obtained powder was further suspended (1% w/v) in a sodium hypochlorite solution (3% v/v) to remove traces of organic material eventually not removed from the first treatment.

### Extraction of the organic components

5 ml of milliQ water, in which 2.5 g of powdered coral skeleton were dispersed, were poured in a 50 cm-long osmotic tube for dialysis (MWCO = 3.5 kDa; CelluSep®, MFPI). The sealed tube was put into 1 L of 0.1 M CH_3_COOH (Riedel–de Haen) solution under stirring. The decalcification proceeded for 72 hr. At the end the tube containing the dissolved OM was dialysed against milliQ water until the final pH was about 6. The obtained aqueous solution containing the OM was centrifuged at 30 g for 3 min to separate the soluble (SOM) and the insoluble (IOM) organic matrix fractions, which were then lyophilized and weighted. The content of OM in the skeleton was gravimetrically determined.

### Characterization of the organic matrix

SDS-PAGE was performed on 12.5% polyacrylamide gel in a vertical slab gel apparatus (Mini-PROTEAN®, Bio-Rad). Different sample volumes were applied for gel lane (10–20 µl). Samples were prepared adding reduced sample buffer 1× (60 mM Tris-HCl pH 6.8; 2% SDS; 2.5% β-mercaptoethanol; 10% glycerol; 0.025% bromophenol blue) and then boiled at 100°C for 5 minutes. The gels ran at a constant voltage of 100 V for 1.5 hr at room temperature. Proteins were detected with Coomassie Brilliant Blue. In the stained procedure, the gel was immersed for 1 hr under shaking in Coomassie Brilliant Blue Staining Solution (0.1% Coomassie Blue R-250 in 1% acetic acid/40% MeOH) and then placed in destaining solutions (25% ethanol and 8% acetic acid) until band became evident. Aminoacid analysis was conducted by a chromatographic technique using an amino acid analyzer. The organic matrix material was weighed, then hydrolyzed at 110°C for 24 hr in 6 M HCl vapor, and analyzed using a Dionex BIOLC amino acid analyzer. Spectroscopic Fourier Transform Infra Red (FTIR) analyses were conducted by using a FTIR Nicolet 380 Thermo Electron Corporation working in the range of wave-numbers 4000–400 cm^−1^ at a resolution of 2 cm^−1^. Disk was obtained by mixing little amounts (<1 mg) of SOM or IOM with 100 mg of KBr and applying a pressure of 48.6 tsi (670.2 MPa) to the mixture using a hydraulic press. UV/Vis analysis was conducted with a Cary UV/Vis 300BIO Varian instrument, in a range between 190 nm e 800 nm, using milliQ water as blank.

### Calcium carbonate overgrowth experiments

Small pieces (about 3 mm) of coral skeleton were place in a Petri dish (d = 5.4 cm) in different orientations, as the overgrowth could not be uniform on all surfaces. The specimens were overlaid with 10.0 mL of 10 mM CaCl_2_ solution. Calcium carbonate crystals were grown for one month. The overgrown specimens were then lightly rinsed with milliQ water, dried and examined in the scanning electron microscope (SEM) after coating with gold.

### Calcium carbonate crystallization experiments

A 30×30×50 cm^3^ crystallization chamber was used. Two 25 mL beakers half-full of (NH_4_)_2_CO_3_ (Carlo Erba) covered with Parafilm 10 times holed and two Petri dishes (d = 8 cm) full of anhydrous CaCl_2_ (Fluka) were put inside the chamber. Microplates for cellular culture (MICROPLATE 24 well with Lid, IWAKI) containing a round glass cover slip in each well were used. In each well, 750 µL of 10 mM CaCl_2_ solutions having Mg/Ca ratio equal to 0, 3 or 5 (CaCl_2_.2H_2_O, Merck; MgCl_2_.6H_2_O, Sigma-Aldrich) were poured. SOM aliquots giving concentration 1.06 mg/mL, 0.44 mg/mL (hereafter reported as *c_s_*) or 0.11 mg/mL were added for each Mg/Ca solution. In other experiments 0.5 mg of IOM (hereafter reported as *c_i_*) were added at each Mg/Ca solution. SOM (*c_s_*) and IOM (*c_i_*) were added in each well in a third set of experiments. The micro-plate was covered with aluminium foil and a hole was made over every well. The experiment proceeded for 4 days. At the end of the crystallization experiment the pH of the solutions in each well was measured. The obtained crystals were washed two times with milliQ water and then analyzed. All the experiments were conducted at room temperature. The crystallization trials of calcium carbonate in the different conditions were replicate at least ten times starting from different batches of organic matrix fractions.

### Characterization of CaCO_3_ precipitates

X-ray powder diffraction patterns were collected using a PanAnalytical X'Pert Pro equipped with X'Celerator detector powder diffractometer using Cu Kα radiation generated at 40 kV and 40 mA. The diffraction patterns were collected within the 2θ range from 10° to 60° with a step size (Δ2θ) of 0.02° and a counting time of 1200 s. FTIR spectra of samples in KBr disks were collected at room temperature by using a FTIR Nicolet 380 Thermo Electron Corporation working in the range of wavenumbers 4000–400 cm^−1^ at a resolution of 2 cm^−1^. A finely ground, approximately 1% (*w*/*w*) mixture of the sample in KBr was pressed into a transparent disk using a hydraulic press and applying a pressure of 48.6 tsi (670.2 MPa). The optical microscope (OM) observations were made with an Leika optical microscope equipped with a digital camera. The SEM observations were conducted in a scansion electronic microscope, Philips SEM-XL20 equipped with a CCD camera after specimens coating with gold and directly in a Phenom™ microscope (FEI).

## Results

**Figure 1:**
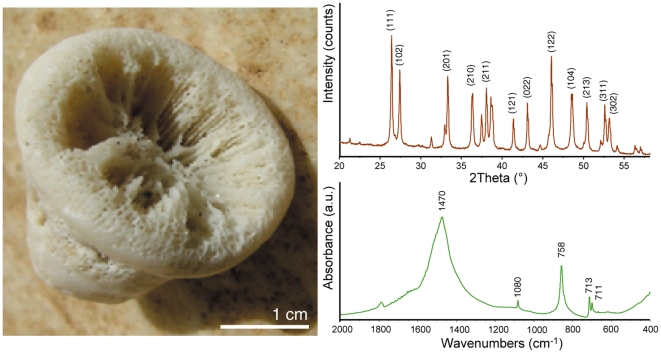
Skeleton of the coral *Balanophyllia europaea*. (A) Digital camera picture of the skeleton of the coral *Balanophyllia europaea* after digestion in a sodium hypochlorite solution to remove the soft organic tissues. (B) X-ray powder diffraction pattern from a powdered coral skeleton sample. Only the characteristic diffraction peaks from aragonite are observable. The main diffraction peaks of the Miller index are indicated according to the reference pattern PDF 98-006-0908 [60]. (C) FTIR spectrum from a powdered coral skeleton sample. Only the typical absorption bands from aragonite were detected. They were assigned as ν_2_ = 858 cm^−1^; ν_3_ = 1470 cm^−1^; ν_4_ = 713 cm^−1^ [42].

XRD and FTIR analyses of the powdered skeletal samples of *Balanophyllia europaea* showed that in the whole skeleton the main calcium carbonate polymorph is aragonite and that calcite sometimes appears in trace amounts. In the bigger skeletons (above 3 g), the ones used in our experiments, only aragonite was present (Fig. 1).

**Figure 2:**
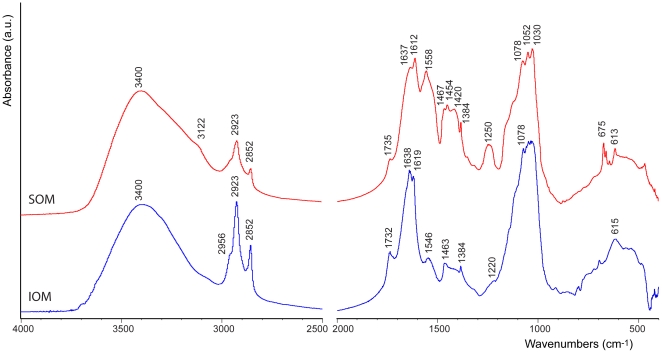
OM characterization. FTIR spectra of intra-skeletal soluble (SOM) and insoluble (IOM) organic matrix from the *Balanophyllia europaea* aragonitic skeleton. Typical absorption bands from protein molecules (around 1600 cm^−1^), polysaccharides (around 1000 cm^−1^) and lipids (around 2900 cm^−1^) are indicated. The absorption due to the polysaccharidic regions appeared stronger than the one due to the proteic regions in both the IOM and SOM spectra.

**Figure 3:**
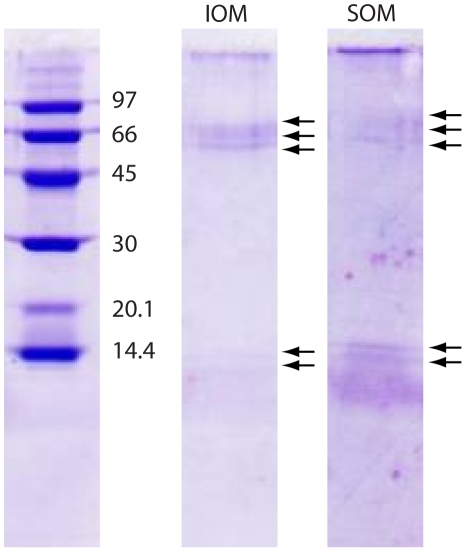
OM characterization. SDS-polyacrylamide gels electrophoresis of intra-skeletal insoluble (IOM) and soluble (SOM) organic matrix extracted from the *Balanophyllia europaea* skeleton. In the first lane the markers are reported. The arrows indicate the major proteic bands.

The percentage of the overall OM in the skeleton, gravimetrically determined, was around 0.3% (*w*/*w*). A water soluble (SOM) and insoluble (IOM) organic matrix fractions were obtained. The mass ratio of SOM and IOM, changed from one experiment to another and was not easy to accurately quantify, however, it was always above 1.5. The chemical-physical characterization of the OM fractions was performed by FTIR spectroscopy, polyacrylamide gel electrophoresis and amino-acidic analyses. In FTIR spectra of both SOM and IOM fractions, typical proteic and polysaccharidic adsorption bands were observable (Fig. 2). The proteic bands of the amide I (1612 cm^−1^ and 1637 cm^−1^ in SOM, and 1619 cm^−1^ and 1638 cm^−1^ in IOM) and II (1558 cm^−1^ in SOM and 1546 cm^−1^ in IOM) were present. The wave-number of the amide I bands at 1637/8 cm^−1^ is typical of a protein in a β-sheet conformation. The amide II band at 1558 cm^−1^ in the SOM and 1546 cm^−1^ in the IOM corresponds to a β-turn and are related to glutamic acid. The relative absorption of the amide II in the SOM fraction was higher than that in the IOM, with respect to the amide I bands; the 1735 cm^−1^ or 1732 cm^−1^ peak is characteristic of the carboxyl group and it is related to aspartic acid. The 1250 cm^−1^ (SOM spectra) and 1220 cm^−1^ (IOM spectra) absorption bands can be attributed to the O-sulphated group or to the amide III [22]–[26]. It was possible to note a shift of both 1558–1546 cm^−1^ and 1250–1220 cm^−1^ peaks from SOM to IOM. The region between 1467–1384 cm^−1^ presented some difference between SOM and IOM fraction: the SOM had peaks at 1467 cm^−1^ (carboxylate group), 1454 cm^−1^ (–CH_2_ or to  = CH_2_ bending), 1420 cm^−1^ (carboxylate group), 1384 cm^−1^ (carbonyl group); all of them were of high intensities and were very well identifiable. The IOM had instead a very weak absorption in that range and only the 1463 cm^−1^ and 1384 cm^−1^ peaks were clearly identifiable. The sugar region (1030–1078 cm^−1^) presented similar pattern of adsorption bands between the SOM and the IOM sample. In the IOM there was only a well resolved peak at 1078 cm^−1^, while in the SOM one there were three evident peaks, 1030 cm^−1^, 1052 cm^−1^ and 1078 cm^−1^. The FTIR spectra also show absorption bands at 2956 cm^−1^, 2923 cm^−1^, and 2852 cm^−1^ that are due to the presence of lipids [41]. The macromolecules comprising the SOM and IOM fractions were also investigated by SDS-polyacrylamide gel electrophoresis. The gel revealed several macromolecular species with molecular masses ranging from ca. 14 to 66 kDa (Fig. 3). Both IOM and SOM fractions were characterized by the presence of the same macromolecular species, gathered around two main molecular weight distributions around 66 kDa and 14 kDa. The relative intensity of the bands in these two regions changed between IOM and SOM, the former being richer of high molecular weight macromolecular species with respect to the latter. The amino acid composition of the proteic regions of IOM and SOM is reported in Table 1. The SOM was characterized by a high content of acidic residues, above 56 mol %: Asx (aspartate or aspargine residues) 50.0 mol %, and Glx (glutamate or glutamine residues) about 6 mol %. On the contrary in the IOM the content of acidic residues was low, about 20 mol%: Asx about 12 mol% and Glx about 8 mol%. The content of hydrophobic residues was higher in IOM than in SOM; in fact Gly, Ala, Val, Ile and Lue represented about 50 mol% and about 30 mol% in IOM and SOM, respectively. In IOM proline was present (about 4 mol%), which was absent in SOM.

**Table 1:** Amino acid compositions (relative mol %) of proteins extracted from the soluble (SOM) and insoluble (IOM) fractions of the *Balanophyllia europaea* skeleton intra-skeletal organic matrix.

	SOM	IOM
Cys	-*	-
Asx	50.0	12.6
Met	-	-
Thr	1.7	4.2
Ser	12.2	9.4
Glx	6.1	8.6
Pro	-	3.7
Gly	18.6	24.3
Ala	4.1	13.2
Val	2.6	4.8
Ile	1.5	4.6
Leu	2.0	5.3
Tyr	-	1.9
Phe	1.2	3.0
His	-	1.2
Lys	-	3.2

*- indicates a not detectable amount.

**Figure 4:**
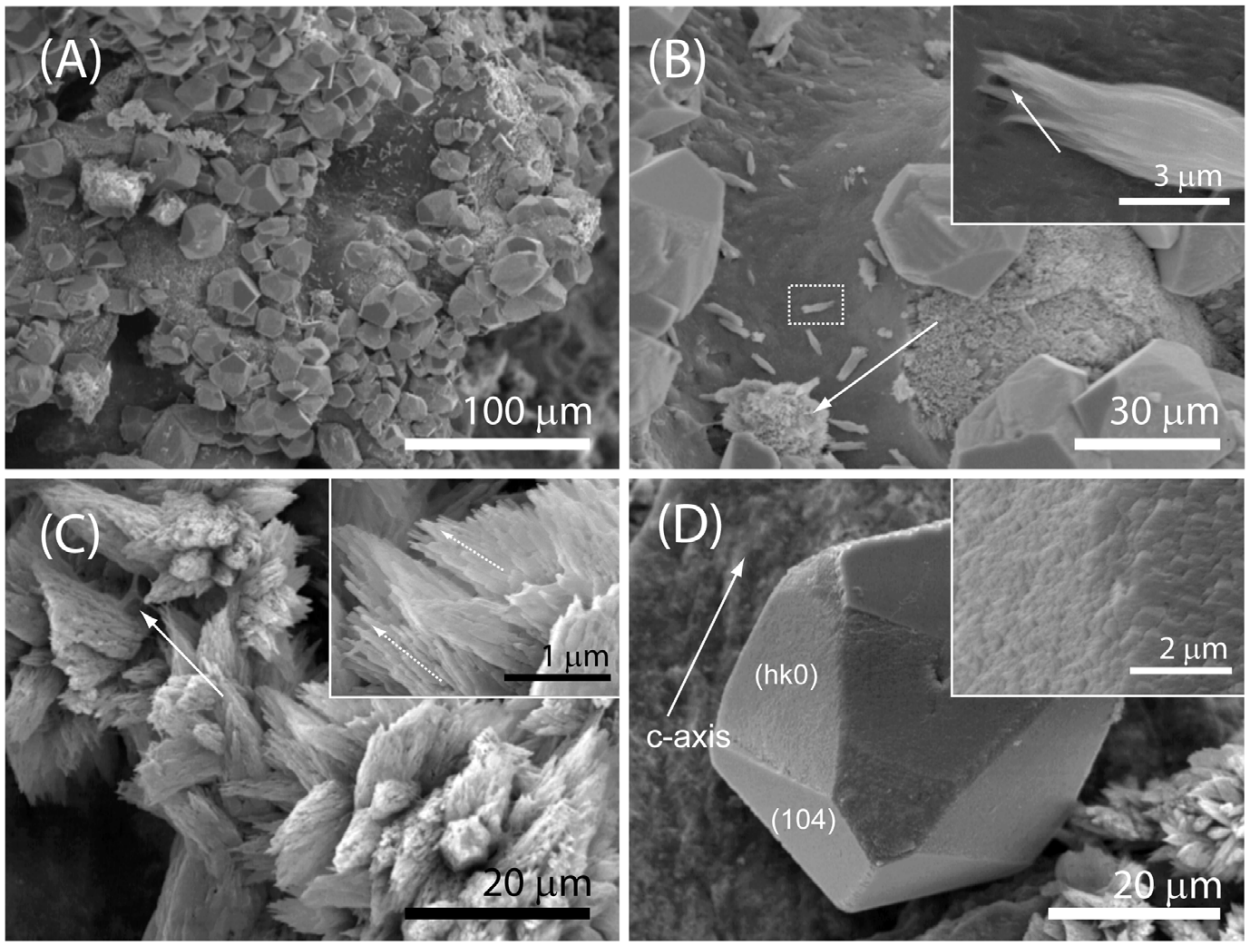
Calcium carbonate overgrowth experiments. (A–D) SEM pictures of a fragment of coral skeleton after the calcium carbonate overgrowth experiment. The overgrowth of calcite and aragonite crystals was observed as shown in (B). In (B) the arrow indicates an aggregate of crystals of aragonite and the dashed square the area illustrated at higher magnification in the inset. The crystals of aragonite appear clustered in bunch of fibers (C), which locally exhibited preferential orientation (arrows in the inset in C). Organic matrix is visible in contact with the crystals of aragonite (arrows in inset in B and in C). The crystals of calcite showed an additional group of {hk0} faces other than the {104} set (D). These new faces showed as smooth surface, typical of interaction with macromolecules (inset in D).

The results of the calcium carbonate overgrown experiments on a coral skeleton fragment are illustrated in figure 4. Overgrowth of aragonite and calcite crystals occurred on the aragonitic skeleton surfaces (Fig. 4-A). The crystals were oriented in different directions, probably reflecting the topography of the substrate. The overgrown of needle like crystals of aragonite occurred in different locations of the skeleton septa as aggregates of different sizes (Fig. 4-B). They usually appeared organized clustered in bunch of fibers which locally exhibited preferential orientation (Fig. 4-C and inset). Organic matrix was observed among the crystals of aragonite (arrows in Fig. 3-B-inset and Fig. 4-C). The overgrown calcite crystals exhibit an additional group of faces other than the {104} set (Fig. 4-D and inset). Measurement of the overgrown calcite crystal faces show that that the new formed faces can be gathered in a family parallel to the crystallographic c-axis. The extension of the new family {hk0} of the crystal faces on the overgrowth calcite crystals changed with the location on the coral skeleton. In rare cases the calcite crystals did show only the {104} cleavage faces.

**Figure 5:**
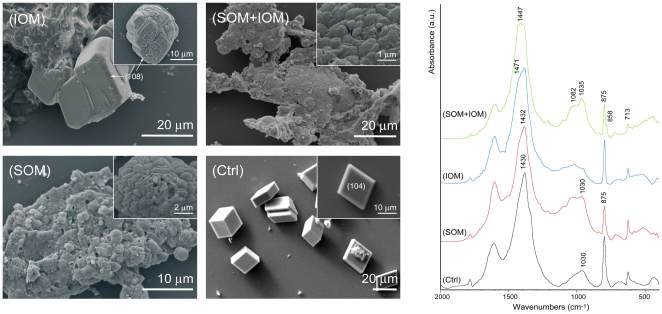
Calcium carbonate crystallization experiments. Crystallization experiments of calcium carbonate from 10 mM CaCl_2_ solution in the absence of additives (Ctrl) and in the presence of soluble organic matrix (SOM), insoluble organic matrix (IOM) or both additives (IOM+SOM). The Miller indexes of calcite faces are indicated. *left* Scanning electron microscope images, the insets show sample details. *right* FTIR spectra of the precipitated. The main absorption bands of calcium carbonate are indicated.

**Figure 6:**
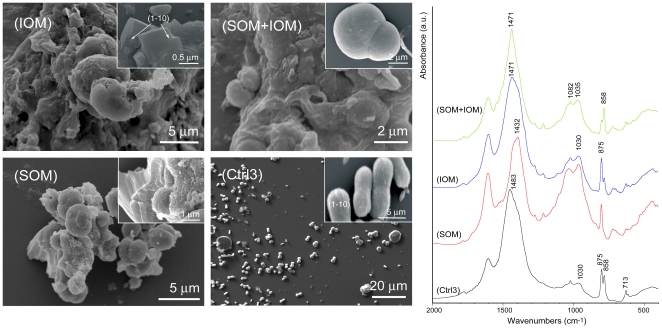
Calcium carbonate crystallization experiments. Crystallization experiments of calcium carbonate from 10 mM CaCl_2_ solution with a Mg/Ca molar ratio equal to 3 (Ctrl3) and in the presence of soluble organic matrix (SOM), insoluble organic matrix (IOM) or both of these (IOM+SOM). The Miller indexes of calcite faces are indicated. *left* Scanning electron microscope images, the insets show sample's details *right* FTIR spectra of the precipitated. The main absorption bands of calcium carbonate are indicated.

**Figure 7:**
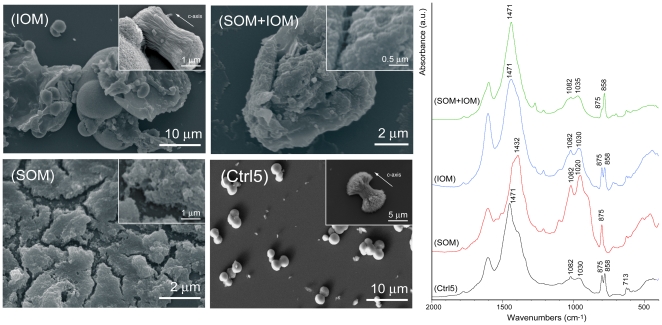
Calcium carbonate crystallization experiments. Crystallization experiments of calcium carbonate from 10 mM CaCl_2_ solution with a Mg/Ca molar ratio equal to 5 (Ctrl5) and in the presence of soluble organic matrix (SOM), insoluble organic matrix (IOM) or both additives (IOM+SOM). The direction of the crystallographic *c*-axis of aragonite is indicated. *left* Scanning electron microscope images, the insets show samples details *right* FTIR spectra of the precipitated. The main absorption bands of calcium carbonate are indicated.

Calcium carbonate was also precipitated in solution in the presence SOM, IOM and/or magnesium ions. The deposited mineral phases were revealed by FTIR measurements and, in some cases, confirmed by XRD (Table 2), while their morphology was observed by optical and electron microscopes (Figures 5, 6, 7). These data summarize the results of more than ten calcium carbonate independent crystallization experiments using different batches of OM components. It is important to state that it was not possible to get a complete control over the crystallization process in the presence of OM fractions. Thus, the presented data represent the general trend of the crystallizations trials. Among the single experiment some difference could be present in the relative amount of polymorphs and the exposition of new crystalline faces in calcium carbonate crystals.

**Table 2:** Crystalline phases precipitated at different amounts of magnesium ions in 10 mM calcium chloride solutions and in the presence of SOM (*c_s_* = 044 mg/mL), IOM (*c*_i_ = 050 mg) or both of them, SOM+IOM (*c_s_* SOM; *c*_i_ IOM).

[Mg^2+^]/[Ca^2+^]	Ctrl*	SOM	IOM	SOM+IOM
**0**	C	C	C	**C** A
**3**	C A	C	**C** A	C A
**5**	**A** C	**C** A	A C	**A** C

A and C indicate aragonite and calcite, respectively. When two phases are obtained the main phase is indicated in bold.#

# The reported data represent the trend observed from more than 10 independent replica of crystallization experiments using different batches of OM fractions. The presence of ACC is not indicated.

*Ctrl indicates mineralization experiment conducted in the absence of organic matrix.

In the absence of additives, neither magnesium ions nor OM components, only the deposition of rhombohedral calcite crystals was observed from a 10 mM calcium chloride solution (Fig. 5-Ctrl). In the presence of a concentration of SOM equal to 0.11 mg/mL (0.25*c_s_*), the precipitation of calcite occurred as in the absence of additives. With a concentration of SOM of 1.06 mg/mL, 2.5*c_s_*, the complete inhibition of the precipitation was observed. In the presence of *c_s_* of SOM an aggregation of the crystals in spherulites that were rhombohedral capped was observed (Fig. 5-SOM inset). These aggregates formed on the surface of a shapeless precipitate (Fig. 5-SOM). The FTIR spectrum of this material showed the characteristic absorption bands of calcite, ν_2_ = 875 cm^−1^; ν_3_ = 1432 cm^−1^; ν_4_ = 713 cm^−1^ [42] plus broad bands at 1030 cm^−1^ and 1082 cm^−1^. These latter two bands could be associated to SOM and to ACC [43], respectively. In the presence of dispersed IOM (*c*_i_ = 0.5 mg) IOM the precipitation of calcite was detected by FTIR. In this case the broad bands around 1050 cm^−1^ showed a weak absorption. Calcite crystals were observed on the IOM and almost conserved the classical rhombohedral morphology (Fig. 4-IOM). The co-presence of SOM (*c_s_*) and IOM (*c*_i_) favoured the precipitation of small crystals that clustered in big aggregates. On their surface, big rounded precipitates formed (Fig. 5-SOM+IOM and inset). The FTIR spectrum of this material showed, together with the absorption bands observed in the presence of SOM, an additional band at 858 cm^−1^, which is characteristic of aragonite (ν_2_) [42]. The second ν_2_ band (844 cm^−1^) of aragonite, related to the substitution of strontium to calcium in the aragonite lattice, was not observed.

A second set of experiments was conducted in the presence of magnesium ions, with Mg/Ca molar ratio equal to 3 (Fig. 6). In this condition the only presence of magnesium ions was able to change the crystal morphology and the polymorphism of the precipitated calcium carbonate (Fig. 6-Ctrl3) with respect to the absence of additives. Indeed, crystals elongated along the c-axis (Fig. 6-Ctrl3, inset) and acicular spherulites (see inset in Fig. 7-Ctrl5) were observed. The corresponding FTIR spectrum showed the typical absorption bands of calcite (ν_2_ = 875 cm^−1^) and aragonite (ν_2_ = 858 cm^−1^), together with the no diagnostic ones. No strong IR absorption was present in the region around 1050 cm^−1^. In the same conditions the addition of *c*_s_ of SOM provoked a strong aggregation of the elongated crystal of magnesium calcite (Fig. 6-SOM and inset), which almost lost the crystalline habitus. The FTIR spectrum of this material did not show the absorption band of aragonite (ν_2_ = 858 cm^−1^), while all those typical of calcite were present, although the band at 713 cm^−1^ (ν_4_) was very weak, in addition to two broad bands centred at about 1030 cm^−1^ and 1082 cm^−1^. In the presence of IOM it was possible to observe the crystals grown on the insoluble matrix substrate and still presenting the typical morphology of magnesium calcite (inset in Fig. 6-IOM). The FTIR spectrum showed typical bands of calcite and aragonite, although acicular spherulites of aragonite were not observed. Also in this case the calcitic ν_4_ (713 cm^−1^) band was very weak compared to the ν_2_ one. The co-presence of SOM and IOM provoked the deposition of surface smooth spherulites (Fig. 6-SOM+IOM and inset). The FTIR spectrum of this material showed the typical absorption bands of calcite (ν_2_ = 875 cm^−1^) and aragonite (ν_2_ = 858 cm^−1^). Broad absorption bands at 1030 cm^−1^ and 1082 cm^−1^ were also present.

The results of crystallization experiments of calcium carbonate from solutions with Mg/Ca molar equal to 5 is illustrated in figure 7. In the absence of SOM and IOM the precipitation of spherulitic aggregates of acicular crystals was mainly observed (Fig. 7-Ctrl5 and inset). The FTIR spectrum showed the typical absorption band of aragonite (ν_2_ = 858 cm^−1^, stronger) and calcite (ν_2_ = 875 cm^−1^, weaker), with weak absorption of the bands at 1030 cm^−1^ and 1082 cm^−1^. The band ν_4_ was centred at 715 cm^−1^ and although weak, was clearly visible. The ν_4_ band of calcite shows a characteristic shift to higher wavenumber when magnesium substitute calcium in the calcite crystal lattice [42]. The addition of *c_s_* of SOM provoked a strong effect on the morphology of the precipitate, which appeared as a layer formed by strongly aggregated micro-units (Fig. 7-SOM and inset). The FTIR spectrum of this material showed the adsorption bands of calcite (ν_2_ = 875 cm^−1^; ν_3_ = 1432 cm^−1^; ν_4_ = 715 cm^−1^) and two strong broad absorption bands centred at 1020 cm^−1^ and 1082 cm^−1^. In the presence of *c*_i_ of IOM the crystals deposited on the surface of the insoluble matrix substrate (Fig. 7-IOM). Some of them were formed by aggregation of acicular crystal, similar to those obtained in the same conditions in the absence of IOM or SOM, but missing the spherulitic shape being preferentially aggregated and aligned in one direction (Fig. 7-IOM inset). The FTIR spectrum showed a similar absorption of the bands at 858 cm^−1^ (ν_2_, aragonite) and at 875 cm^−1^ (ν_2_, calcite). The bands at 1030 cm^−1^ and 1082 cm^−1^ were also clearly visible. Figure 7-SOM+IOM shows the results of the precipitation in the presence of SOM and IOM. No typical crystalline habits were visible in the precipitate in which the mineral phase appeared completely embedded in the OM. The FTIR spectrum of this material showed the presence of a strong absorption band due to aragonite (ν_2_ at 858 cm^−1^) and a weak one due to calcite (ν_2_ at 875 cm^−1^). Weak absorption was also associated to the bands at 1030 cm^−1^ and 1082 cm^−1^.

**Figure 8:**
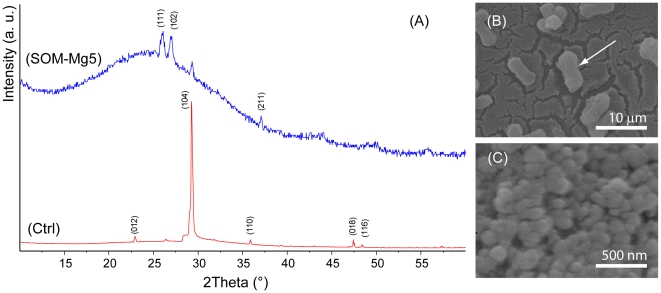
Calcium carbonate crystallization experiments. (A) X-ray powder diffraction patterns of the precipitates obtained from a 10 mM calcium chloride solution (Ctrl), from a 10 mM calcium chloride solution containing SOM and magnesium ions in Mg/Ca molar ratio equal to 5 (SOM-Mg5). In (Ctrl) only calcite is present, while in (SOM-Mg5) a mixture of calcite and aragonite is present. Moreover, in (SOM-Mg5) the broad band around between 20° and 40° suggests the presence of amorphous material. The calcite diffraction peaks, (012), (104), (110), (018) and (116), were indexed according to the reference pattern PDF 98-000-5342 [61], while the aragonite diffraction peaks, (111), (102) and (211), were indexed according to the reference pattern PDF 98-006-0908 [60]. (B and C) SEM pictures of the precipitate obtained from SOM-Mg5. Crystals of magnesium calcite (see arrow in (A)) and aragonite deposited on, and into, a jagged layer of ACC. In (B) is shown a magnification of the ACC layer, in it small particles of about 100 nm in diameter are visible.

The FTIR spectra of the mineral phases obtained in the presence of *c_s_* of SOM and magnesium ions suggested that ACC could be present in the precipitate. These precipitates were also investigated by XRD. In figure 8A, as example, the XRD patterns of the precipitate obtained in the presence of *c_s_* of SOM and Mg/Ca molar ratio equal to 5 and of the material precipitated in the absence of additives are reported. In the diffraction profile (SOM+Mg5) a broad band spanning between 20° and 40° of 2theta was present, which is typical of amorphous material. In figure 8B–C SEM pictures of the precipitate from the SOM+Mg5 solution are shown. ACC forms a jagged layer on/into which crystals of magnesium calcite and aragonite are deposited (Fig. 8B). The ACC layer is formed by the random assembly of particles of about 100 nm in diameter (Fig. 8C).

## Discussion

In this research the intra-skeletal OM associated to the whole skeleton, COC and fibrous regions, of a solitary Mediterranean coral, *B. europaea*, was studied. It represented about 0.3% (*w*/*w*); this value is far lower than the amount detected by means of thermo-gravimetric analyses, about 1.5% (*w*/*w*), in coral skeleton from other species [44]. The different evaluation method could be one of the reasons for this difference. Moreover, in the used OM extraction protocol a fraction OM is degraded by the powered skeleton treatment in the NaClO solution and the OM low molecular weight components are lost during the dialysis process. OM fractions, water soluble (SOM) and water insoluble (IOM), were investigated, differently from the previously reported studied which mainly focused on the soluble OM fraction [22], [23], [25], [27], [45]. This distinction allowed evidencing some important features. An important observation concerns the relative amount of SOM and IOM that does not appear constant, but changes from one experiment to another. A possible explanation could be the tendency of these macromolecules to undergo to partial denaturation, once extracted from the mineral phase, and then to aggregate when in solution [46]. Indeed it was observed an increase of the insoluble fraction as the amount of extracted material increased (data not reported). The gel electrophoresis of SOM and IOM showed that the same macromolecules, as molecular weight, are present in both fractions. This indicates that the solubility of the two fractions is probably related to their degree of cross-linking and/or association in water. Thus, it is possible to infer that in the studied coral the same macromolecules are assembled in different ways, both to build the matrix framework and to exert their function of control over the mineral deposition. This does not match with what reported for mollusc shells, where a clear compositional distinction occurs between the soluble and the insoluble macromolecules that compose the OM [1]. Moreover, the gel electrophoresis showed that the OM macromolecules were mainly gathered in a high and a low molecular weight families. The SOM fraction, richer of low molecular weight macromolecules (around 14 kDa), showed an amino acid composition characterised by a high content of acidic residues, above 50 mol %, mainly represented by Asx. On the contrary the IOM fraction, richer in high molecular weight macromolecules (around 66 kDa), is low acidic and contains more hydrophobic residues. Interestingly, the IOM contains proline, an amino acid usually associated to fibrous proteins. The amino acid analyses conducted on the soluble extract from several coral skeleton specie are in agreement with the above observation e.g. [22], [23], [25], [27], [45]]. SOM and IOM are mainly glycoproteins in which the glycosidic regions represent their major component, as shown by the FTIR spectra (Fig. 2). The absorption bands associated to the glycosidic regions have a similar pattern, suggesting that the sugars have the same structure in SOM and IOM. This observation matches with the above considerations, which suggest that IOM and SOM are made of the same macromolecules, but with a different distribution between two main families. A significant content of lipids was also detected by FTIR spectroscopy in both SOM and IOM (Fig. 2). They could have an influence in the mineralization process. They could be involved in the stabilization of ACC as final or transient phase. In particular phospholipids could play a double role in stabilizing ACC through the interaction with phosphate groups and isolating the mineral phase through the formation of vesicles [41], [47].

A key to understanding the mechanisms and functions of biological macromolecules in mineralized tissues is the recognition between the macromolecules and the crystal faces and/or the stabilization of unfavoured mineral phases [1]–[3]. Overgrown experiments allow to study specific crystal-macromolecules interactions [48]. In these experimental conditions the acidic macromolecules-crystal interactions occur under conditions that minimally affect the macromolecules. Local dissolution of skeletal surface and release of acidic macromolecules into the overgrowth microenvironment must be involved in the mechanism of expression of new crystal faces and/or stabilization of unfavoured mineral phases. Dissolution and crystallization, which take place in the same microenvironment, may be due to the lower thermodynamic stability of the biogenic mineral, which must be due to the presence of occluded acidic macromolecules. In overgrowth experiments, although the macromolecules are not in a native state, they are certainly more conducive to preserving their structure that after isolation and separation procedures, which often result in macromolecule denaturation. However, not all the intra-skeletal acidic macromolecules could be released by the skeletal elements or be able to interact with the overgrowing phases. The presence of overgrowth aragonite on the coral skeleton may indicate a capability of the organic matrix component to stabilize this polymorph, even in the absence of magnesium ions. The fact that aragonite was observed only on the surface of the skeletal elements and in contact with the OM suggests a role of IOM in aragonite stabilization. The calcite crystals overgrown on coral skeleton showed crystal faces {hk0} other than those of the stable cleavage {104} habit. These faces develop as a result of selective re-adsorption of the soluble acid macromolecules released from the coral skeleton, as also suggested by their smoothness [48]. Calcite crystals showing the new crystal faces were also observed in proximity of the coral skeleton and not in contact with it, indicating a diffusion of SOM components in solution. A different expression of the new crystal faces on the overgrowth crystals depending of the location on the coral skeleton was also noted. An explanation could be that the release of acidic macromolecules form any surface other than the plane of intercalation is hampered. This factor will cause differences in acidic macromolecules availability in various directions, depending on their orientation relative to the surface of the crystal. The above observations from the overgrowth experiments clearly suggest that the OM components from the coral skeleton are able to influence the calcium carbonate precipitation. Thus, calcium carbonate precipitation experiments were also conducted to evaluate the SOM and IOM fractions influence on the mineralization process. This was done using an *in vitro* model system of the crystallization process [49]. This model system is far from the real biological environment, but is able to simulate the mineralization process by increasing concentration of carbonate ions [1]–[3] and was widely used in all the recent literature. The experiments were conducted using as additives SOM, IOM and magnesium ions. The SOM was studied at different concentrations. It was observed that a low concentration of SOM (0.25*c_s_*) does not affect the precipitation of calcium carbonate, while a high concentration (2.5*c_s_*) provoked a complete inhibition of precipitation. A concentration of SOM, *c_s_* was chosen with the criterion to compromise between the inhibition of precipitation and the need to assess its influence on the precipitation process. Interestingly, a *c_s_* concentration of SOM favoured the precipitation of calcite, even in conditions where in its absence aragonite precipitated, i.e. from solution having Mg/Ca molar ratio equal to 3 or 5 (Table 2). This effect could be due to a preferential interaction with magnesium ions that may reduce their activity in solution. Indeed, it is reported that the precipitation of aragonite from solutions containing magnesium ions is due to an inhibition of the precipitation of calcite; magnesium ions poison crystal nuclei of calcite and stop their growth [28]. This mechanism of preferential interaction of acidic macromolecules with magnesium ions was also proposed to explain the switch off of ACC stabilization done by some specific aspartate rich macromolecules [11]. Another possibility is that the SOM inhibits the precipitation of aragonite, interacting with some specific crystalline planes and stopping the growth of the crystals in a process mediated by magnesium ions, as observed to occur in calcite as a consequence of mineral-macromolecules interaction [50]. This latter hypothesis is further supported by the observation that in the presence of SOM the morphology of the crystals, mainly the calcitic ones, is strongly modified (Figs. 5SOM and 7SOM). It was demonstrated that aragonitic macromolecules are able to interact with calcite crystals in analogous ways to what may occur with biogenic aragonite crystals [49]. Moreover, in the presence of SOM, and in minor extent in the presence of IOM, ACC appears to be present in the precipitates. This is indicated by the FTIR strong absorption band around 1080 cm^−1^, which could also be due to the SOM itself, by the weak peak at 713 cm^−1^ [42] and by the XRD data. In fact, the precipitation of ACC is favoured by magnesium ions and molecules that inhibit calcium carbonate crystalline phases precipitation [29], [51], [52]. Interestingly the ACC precipitate forming aggregates of spheres showing a diameter of about 100 nm. The size of these particles, above the critical size (70 nm) of those formed in bulk, requires their stabilization by components of the organic matrix [53]. The existence of similar particles of ACC was observed to occur *in vivo* in molluscs. It was suggested that these particles had the role of calcium and carbonate ions reservoirs to feed the growing crystals of calcium carbonate [46], [54]. This may suggest that AAC could be also involved in the precipitation of aragonite in corals, analogously to what observed in molluscs. However, differently from the molluscs, no direct observation of ACC in corals was so far reported [43], [55]–[57]. A precipitation mechanism involving ACC similar to that observed during shell molt of crustaceans may occur also in corals. There a magnesium ions-aspartate-based calcium carbonate crystallization switch from ACC into calcite was demonstrated to occur. The ACC stabilizing role of magnesium ions is switched off by Asp-rich proteins. Thus, these proteins favour the polymorphic transition from ACC to a crystalline phase [11]. Although the OM macromolecules from coral skeleton have a high content of aspartate, this destabilization of the ACC has not been observed and the experimental data suggest an opposite effect. However, the involvement order of magnesium and aspartate is critical to compose the crystallization switch. No switch is observed if the ACC is first stabilized by aspartate, followed by the addition of magnesium ions. Since in the presented experiments there is the co-presence of aspartate rich acidic macromolecules and magnesium ions, the absence of a crystallization switch does not surprise. Nevertheless, it is conceivable that the biological system controlling the aragonite deposition in corals may use magnesium to stabilize ACC and the acidic macromolecules to address specifically the crystallization process.

The presence of IOM had a minor effect on the polymorphism of precipitated calcium carbonate. The polymorphic distribution as a function of the Mg/Ca molar ratio in solution was similar to that observed in its absence (Table 2). However, the morphology of the crystal was affected, suggesting that an interaction between the macromolecules released by the IOM and specific crystalline planes of calcite and aragonite occurred. The calcite crystals showed additional crystalline planes ({018} in the absence of magnesium ions and {1–10} in the presence of magnesium ions) with respect to the {104} rhombohedra obtained in the absence of additives. The aragonite crystals appeared cut along their main axis, c-axis, as a result of the presence of IOM and magnesium ions, suggesting an interaction between IOM macromolecules and {001} crystalline planes of aragonite, mediated by the presence of magnesium ions. This observation may have some implication with the zonal distribution of magnesium ions in the fibrous aragonite region of corals that could be mediated by OM molecules [50], [58].

The precipitation experiments with the co-presence of IOM and SOM in the media should be the ones that better may symbolize the *in vivo* system, being the OM components similar to those present in the coral skeleton. In this condition, even the absence of magnesium ions, the precipitation of aragonite, as minor phase, together with calcite was observed. It is important to note that the only presence of the single fractions of the OM, SOM or IOM, was unable to induce the precipitation of aragonite. The presence of magnesium ions strengthens the capability of the IOM+SOM mixture to favour the precipitation of aragonite, and at a Mg/Ca molar ratio equal to 5 aragonite is the main phase present. The presence of magnesium ions also influences the morphology of the precipitates and when increasing its concentration the precipitated mineral lost crystalline features and was completely entrapped in the dry OM (or xerogel). This outcome suggests that the mineralization process occurs in a gelling environment.

The fact that both, IOM and SOM, are necessary to exert a high influence on calcium carbonate crystal morphology and polymorphism was already reported for mollusc shells, since the acidic macromolecules extracted from aragonitic layers are able to precipitate aragonite only when absorbed in a specific gelling substrate made of chitin and silk fibroin or in synthetic analogous [7], [8], [10]. The precipitation of aragonite in the presence of the OM from coral may also occur in a gelling environment. Indeed, both IOM and SOM components are rich of polysaccharides that usually have strong tendency to entangle giving a gelling structure [5], [59] on which the acidic macromolecules, mainly from SOM, could adsorb and exert their control on the calcium carbonate precipitation.

This research represents, to our knowledge, the first study on the influence of the intra-skeletal OM from coral skeletons on *in vitro* precipitation of calcium carbonate. The OM contains two main families of acidic glycoproteins that when extracted aggregates giving a water soluble and an insoluble fraction. The soluble fraction strongly interacts with aragonite and calcite crystals and favours the precipitation of calcite or the inhibition of aragonite. The co-presence of both fractions allows the co-precipitation of aragonite with calcite, even in the absence of magnesium ions, and strongly favours the precipitation of aragonite in the presence of magnesium ions. Thus, this research, although does not resolve the controversy of the weight between environmental or biological control on the deposition of calcium carbonate in coral skeletons, sheds a light on the role of the OM, which appears to be regulated by magnesium ions. In conclusion on the base of our results we may safely conclude that the OM composition and magnesium ions influence the fine scale characteristics of the crystals of which the coral skeleton is constructed.
